# Metabolic imbalance in affective disorders

**Published:** 2013-03-25

**Authors:** M Ladea, CM Barbu, DP Rosu

**Affiliations:** "Prof. Dr. Alexandru Obregia" Clinical Hospital of Psychiatry, Bucharest

**Keywords:** diabetes, depression

## Abstract

**Introduction:** Depression is the most frequent mental disorder encountered in all medical services. Multiple studies have shown that depression may predict the onset of different conditions, such as heart disease, diabetes, stroke and many other. The relation between depression and diabetes is still unclear.

**Materials and methods:** In this study we evaluated patients with both major depressive disorder and type 1 or 2 diabetes, and observed the evolution of depressive and diabetes symptoms under adequate treatment.

This observational, naturalistic study included 43 patients admitted in a psychiatric ward of "Alexandru Obregia" Clinical Hospital, Bucharest, diagnosed with Major Depressive Disorder (MDD) and Diabetes mellitus (DM) type 1 or 2. All patients received antidepressant and antidiabetic treatment.

**Results: **The majority of patients were women (60.5%), and the mean age was 49.7 years. Average hospitalization period was 23 days, with longer period of hospitalization of patients with DM type 1. Patients had severe depression. Mean value of fasting glycemia at admission was of 174 mg / dl, but it decreased at discharge, in paralel with the amelioration of depression.

**Conclusions:** The depression associated with DM type 1 is more severe. These patients require higher doses of antidepressants and longer hospitalization period. Amelioration of depression seems to have a positive impact on the blood sugar level of depressed patients with diabetes.

## Introduction

Depression is probably the most frequent mental disorder encountered in all medical services. Recent studies have reported that the lifetime prevalence of a major depressive disorder in the United States was of 16.2% [**1**], whereas the lifetime prevalence in Europe was of 14% [**2**]. According to the World Health Organization (WHO), depression is responsible for the greatest proportion of burden associated with non-fatal health outcomes and account for approximately 12% of total years lived with disability [**3**]. According to the World Health Organization estimations, in 2020 depression will be the second cause of morbidity in the world [**3**]. The effect of depression on the quality of life might be greater than the influence of diabetes [**4**]. 

Studies have shown that depression may be a risk factor for different conditions, such as heart disease, diabetes, stroke and many other [**5**].

 Diabetes is one of the leading causes of death and disability worldwide, with 150 million cases estimated currently and 300 million cases estimated by 2025 [**6**]. Based on etiology, diabetes is divided into four categories: type 1, type 2, gestational and the other specific types due to different causes [**7**]. In general, diabetes affects multiple aspects of quality of life, including physical, emotional and social well-being [**8**].

 The relation between depression and diabetes is still unclear. It has been suggested that each disorder is a risk factor for the development of the other and that both may share some common pathophysiological mechanisms and that there is strong evidence for a bidirectional relationship between diabetes and depression [**9,10**]. 

 The prevalence of clinical depression and presence of elevated depressive symptoms are higher among persons with diabetes compared with the general population. These associations may be related to increased risk of depressive symptoms in individuals with diabetes, increased risk of type 2 diabetes in individuals with depressive symptoms, or both [**11,12**].

 The other studies are centered on a different hypothesis and suggest that depression is associated with diabetes and the different biological processes represent the link between them. These include the association between depression and insulin resistance, the involvement of hypothalamic-pituitary-adrenal axis, the innate immune response and the involvement of autonomic nervous system [**13-15**].

Insulin resistance is defined as decreased sensitivity of the peripheral insulin receptors to the action of insulin. Depression is frequently associated with a sedentary life-style, which may lead to insulin resistance. Individuals with obesity have an elevated risk to develop depression and they also have an increased risk of developing insulin resistance [**16-18**].

 Insulin resistance is also a determinant of free fatty acids in the blood, which are in turn important in tryptophan metabolism and brain serotonin concentrations [**13**]. Serotonin, which is derived from tryptophan, is involved in the etiology of depression.

 The hypothalamic-pituitary-adrenal axis (HPA) activates the sympathetic nervous system, which increases catecholamines, stimulates the immune response and decreases neurovegetative functions. The activation of HPA is reflected in the following metabolic processes: gluconeogenesis, insulin resistance and glycogenolysis [**13,19,20**]. 

 The innate immune system releases from the macrophages and other cells pro-inflammatory cytokines: interleukin IL-6, IL-1 and tumor necrosis factor TNF-α. The macrophages accumulated in the fat tissue produce pro-inflammatory cytokines. Cytokines may determine behavioral changes, such as depressive symptoms. Production of pro-inflammatory cytokines is also associated with pancreatic β-cell apoptosis, reduced insulin secretion, insulin resistance and onset of type2 diabetes [**13,20,21**].

 Depression affects about 20% to 25% of diabetic patients, nearly twice as many as the general medical population. Depression is common in both type 1 and type 2 diabetes and has significant effects on the course and outcome of this medical condition. Known to impair overall functioning and quality of life, depression has additional importance due to its association with poor compliance with diabetes treatment, poor sugar level control, and an increased risk for micro- and macrovascular disease complications. 

 The interaction of diabetes and depression has been found to be synergistic, predicting greater mortality, greater incidence of both macrovascular complications and microvascular events and greater incidence of functional disability in the activities of daily living [**22,23**].

 Despite its relevance to the course of diabetes, depression is recognized and treated in approximately one third of cases. Criteria-based diagnostic systems are sensitive and valid methods for detecting depression in diabetes even though unstable diabetes may produce some symptoms of depression [**24**]. 

Conventional antidepressant management strategies are effective and the regimen should be tailored to the individual patient. Diabetes and coexisting depression have higher all-cause mortality relative to non-depressive diabetic patients. The available literature suggests that clinically significant levels of depressive symptoms are associated with a range of poorer self-care behaviors including adherence to diet, exercise and prescribed medications [**25**].

## Materials and methods

Depression can have a significant influence on the outcome of diabetes. On the other hand, diabetes can influence the evolution of a depressive episode. In some cases, diabetes may be the trigger for a depressed mood, which later can lead to a major depressive disorder. 

 In this study, we evaluated the patients with major depressive disorders and diabetes, control of diabetes and evolution of depressive and diabetes symptoms under antidepressant treatment.

 This observational, naturalistic, study included 43 patients admitted in a psychiatric ward of “Alexandru Obregia" Clinical Psychiatric Hospital Bucharest, Romania, diagnosed with Major Depressive Disorder (MDD) according to DSM-IV-TR and Diabetes mellitus (DM) type 1 or 2. The age ranged from 30 to 76 years old and included female and male patients. 

 The depressive symptoms were assessed by using Montgomery Åsberg Depression Rating Scale (MADRS) at admission and discharge. MADRS is a reliable scale for depression in patients with comorbidities. DM was evaluated by monitoring the morning sugar level and glycosylated hemoglobin A. The diabetic complications were assessed through clinical evaluations. 

 All the patients received antidepressant treatment and antidiabetic treatment (methformin or insulin) according to a specialist prescription.

## Results

The majority of the patients were women (60.5%) and the mean age was 49.7 years. The average hospitalization period was of 23 days and the mean score of MADRS at admission was 36, which confirmed a severe depression. Average values of morning sugar level at admission were 174 mg/dl and then decreased during the hospitalization, in parallel with the improvement of depressive status.

**Fig. 1:**
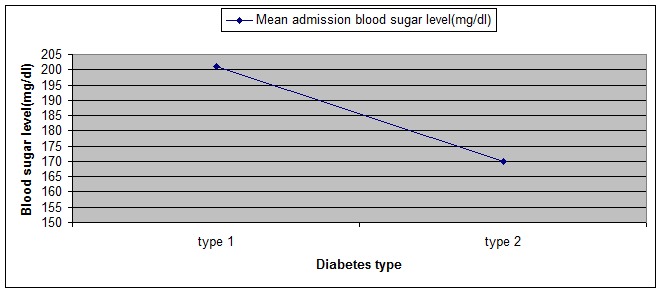
Morning sugar levels at admission according to diabetes type

**Fig. 2:**
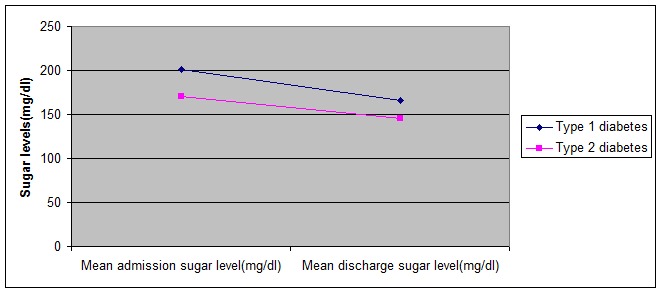
Mean sugar levels at admission and discharge according to diabetes type

At admission, the mean sugar level was significantly higher in type 1 diabetes (**Fig. 1**). With adequate antidepressant and antidiabetic treatment, the mean sugar level of both types of diabetes decreased until the discharge. With adequate treatment, the mean sugar levels at discharge of type 1 diabetes remained higher compared to type 2 diabetes (**Fig. 2**).

**Fig. 3:**
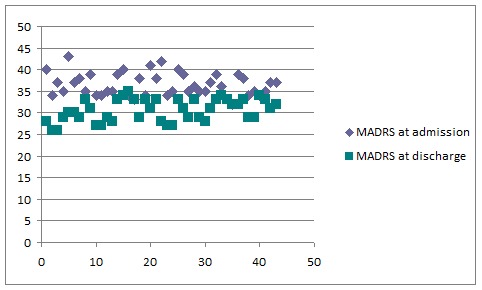
MADRS scores at admission and discharge

The MADRS scores at admission and discharge for all the patients are illustrated in **Fig. 3**. 

 The prevalence of DM type 1 was of 16%. We observed that in patients with DM type 1, the depressive symptoms were more severe than in patients with DM type 2. In both types of diabetes, the MADRS scores at admission were significantly higher than the discharge scores (**Fig. 3**), which indicates a favorable evolution.

**Fig. 4:**
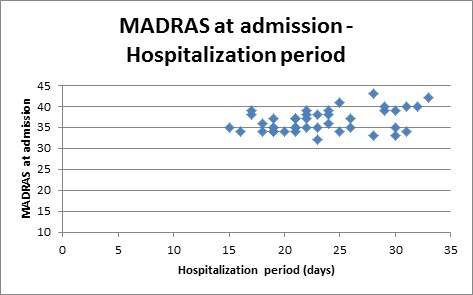
Patients distribution according to hospitalization period and MADRS at admission

## Conclusions

The depression associated with DM type 1 was more severe in the patients we have observed. For these patients higher doses of antidepressants and more hospitalization days were necessary. Adequate antidepressant treatment seems to have a positive impact on the blood sugar levels. 

 Concurrent depression is associated with a decrease in the metabolic control, poor adherence to medication and diet regimens, a reduction in quality of life, and an increase in health care expenditures. 

 Commentaries

 The successful management of diabetes and depression requires not only the intensification of the efforts in the therapy of depression as well as in medical therapy of diabetes, but a much higher level of prolonged cooperation between different disciplines for optimal results for the patient [**26**]. 

 More frequent controls, better follow-up and psychotherapeutic techniques such as cognitive behavioral therapy can be useful to create an effective treatment plan for those with diabetes and depression [**26**].
